# Prevalence, incidence and bothersomeness of urinary incontinence between 6 weeks and 1 year post-partum: a systematic review and meta-analysis

**DOI:** 10.1007/s00192-021-04877-w

**Published:** 2021-06-17

**Authors:** Heidi F. A. Moossdorff-Steinhauser, Bary C. M. Berghmans, Marc E. A. Spaanderman, Esther M. J. Bols

**Affiliations:** 1 Faculty of Health, Medicine and Life Sciences, Department of Epidemiology, Care and Public Health Research Institute (CAPHRI), Maastricht University, P.O. Box 616, 6200 MD Maastricht, The Netherlands; 2 Pelvic care Center Maastricht, CAPHRI, Maastricht University Medical Centre (MUMC+), Maastricht, The Netherlands; 3 Department of Obstetrics and Gynaecology, MUMC+, Maastricht, The Netherlands

**Keywords:** Bother, Incidence, Post-partum, Prevalence, Systematic review, Urinary incontinence

## Abstract

**Introduction and hypothesis:**

Urinary incontinence (UI) is a common complaint for post-partum women. Reported prevalence and incidence figures show a large range due to varying study methodology. The crude prevalence of post-partum UI may differ when accounting for bother. Precise prevalence and incidence figures on (bothersome) UI are of relevance for health care providers, research planning, and policy makers. Therefore, we conducted a systematic review and meta-analysis to investigate the prevalence and incidence of UI in post-partum women in the Western world for relevant subgroups and assessed experienced bother in relation to UI.

**Methods:**

Observational studies, published between January 1998 and March 2020 and reporting on prevalence and incidence between 6 weeks and 1 year post-partum, were included, regardless of type of UI or setting. We used a random effects model with subgroup analyses for post-partum period, parity and subtype of UI.

**Results:**

The mean (weighted) prevalence based on 24 included studies, containing a total of 35.064 women, was 31.0%. After an initial drop in prevalence at 3 months post-partum, prevalence rises up to nearly the same level as in the third trimester of pregnancy at 1 year post-partum (32%). Stress UI (54%) is the most prevalent type. UI prevalence is equal among primi- and multiparous women. Experienced bother of UI is heterogeneously assessed and reported to be mild to moderate.

**Conclusions:**

Post-partum UI is highly prevalent in women in the Western world. After an initial drop it rises again at 1 year post-partum. Experienced bother is mild to moderate.

**Supplementary Information:**

The online version contains supplementary material available at 10.1007/s00192-021-04877-w.

## Introduction

Urinary incontinence (UI) is the complaint of involuntary loss of urine [1]. The main subtypes of UI are stress (S) UI, urgency (U) UI and mixed (M) UI. SUI is leaking urine when coughing or sneezing [1]. SUI is more common in younger women [2]. Pregnancy and vaginal delivery are well-documented risk factors for developing UI [3–5]. Seventy-three percent of women with UI 3 months post-partum still report UI at 6 years post-partum [6]. In general, UI prevalence and incidence rise with ageing [7]. Women often experience UI as embarrassing and humiliating, resulting in loss in quality of life [8]. UI also causes considerable socio-economic costs [9, 10].

The prevalence and incidence of UI in the post-partum period are widely studied. However, these prevalence and/or incidence figures vary greatly throughout published reports, depending on local setting, case definitions applied, recruited population (period post-partum and parity) and study methodology [11, 12]. A systematic review on the prevalence of post-partum UI and the relation to the mode of delivery was published in 2010 [13]. At that time, studies hardly reported on bother. In 2017, the International Consultation on Incontinence (ICI) recommended that prevalence numbers should be accompanied by the experienced bother [14], as there are indications that the prevalence of bothersome UI is lower than the crude UI prevalence [14]. As women with bothersome UI tend to seek more help [15], health professionals, policy makers and researchers need reliable prevalence numbers to specify the health problem UI causes and to help set priorities and assist in planning the management of UI.

Therefore, the primary aim of this systematic review and meta-analysis was to examine the pooled overall prevalence and incidence of UI between 6 weeks and 1 year post-partum in the general population of the Western world, specified for relevant subcategories (period post-partum, parity, type of UI, frequency and amount). A secondary aim was to provide an overview of the assessment methods and outcomes for bother in relation to UI as used in included studies.

## Methods

The MOOSE statement for reporting systematic reviews and meta-analyses was followed [16]. The research protocol was published in the PROSPERO database (registration number CRD42018111991).

### Search strategy

We performed a systematic review and meta-analysis of observational studies (cross-sectional and cohort studies) reporting on the prevalence and incidence [17] of UI after delivery and experienced bother. We searched the electronic databases of PubMed, EMBASE and CINAHL. All included articles were reference checked. Titles and/or abstracts of studies retrieved using the search strategy and those from additional sources were screened independently by two reviewers. Full texts of potentially eligible studies were retrieved and independently assessed for eligibility by two review team members. Any disagreement on eligibility was resolved through discussion with a third reviewer.

We used the following search terms to search all databases: postpartum, post-partum, post partum, peripartum, peri-partum, peri partum, primiparous, multiparous, multigrav*, multipar*, urinary incontinence, urine loss, leaking urine, incontinence, prevalence, incidence, epidemiology, frequency, bothersomeness, bother*, quality of life and hindrance. In the Appendix the complete search strategy for PubMed is provided. This search string was adapted for use in the other databases.

### Eligibility criteria

Observational studies published between January 1, 1998, and March 1, 2020, in Dutch, English, German and French were included. All studies examining prevalence and/or incidence of UI from 6 weeks to 12 months post-partum among adult primi- and multiparous women in the Western world, regardless of type of UI and setting, were of interest. Six weeks post-partum was chosen to ensure a large proportion of the sample had recovered physiologically from the delivery. Outcomes of interest were prevalence and/or incidence of (bothersome) UI. Exclusion criteria were: articles not available in full or not reporting an overall UI prevalence and/or incidence of any frequency, studies examining only twin pregnancies and studies originating from non-Western countries. The latter criteria were chosen for the purpose of homogeneity in population characteristics. When articles did not report a prevalence figure or response rate, an attempt was made for estimation from the information provided. Throughout this article we use the term bother (in relation to UI) as an umbrella term for related constructs [impact on daily life or quality of life (QOL)].

### Study selection

Titles and/or abstracts of studies retrieved using the search strategy and those from additional sources were screened independently by two reviewers (HM and EB) to identify studies that potentially meet the inclusion criteria outlined above. The full texts of these potentially eligible studies were retrieved and independently assessed for eligibility by these two reviewers. Any disagreement on eligibility was resolved through discussion with a third reviewer (BB). All the included articles were reference checked.

### Data extraction and risk of bias

Information on each study was extracted in a standardized data extraction form, based on the Cochrane Public Health Data Extraction and Assessment template [18]. To assess the risk of bias, the Joanna Briggs critical appraisal tool for studies reporting prevalence data was used [19, 20]. The checklist consists of nine questions, with the response options yes, no, unclear or not applicable. Overall risk of study bias was rated as low (defined as 8–9 criteria answered as ‘yes’), moderate (4–7 criteria answered as ‘yes’) or high risk (≤ 3 criteria answered as ‘yes’). The response option not applicable (occasionally scored in criteria 5) was considered to be a ‘yes’. Two reviewers (HM, EB) extracted data independently. Inconsistencies were identified and resolved through discussion including a third author (BB) if necessary.

Characteristics regarding measurement instruments for bother were extracted in a separate standardized extraction form. The form contains items such as measurement instrument, related construct and measurement results.

### Summary measures, statistical analyses and heterogeneity

We used a random effects model to pool the inverse variance (IV) weighted prevalence of UI in individuals to avoid undue influence on the summary estimate from smaller and less precise studies or studies with a very small prevalence. Pooled prevalence and incidence values were reported with 95% confidence intervals (CI). The degree of heterogeneity was determined by the I^2^ statistic, with I^2^ > 75% labelled as high heterogeneity [21].

Prevalence was studied by subgroup [post-partum period (6 weeks, 3, 6, 9 and 12 months), type and frequency of UI, and parity (primi- and multiparous)] as this might explain why studies show varying prevalence figures. Studies reporting on a post-partum period other than the five established periods are classified in the closest post-partum period. Moreover, studies reporting a period prevalence (e.g. 9–12 months post-partum) are classified in the upper range of the period prevalence (i.e. 12 months), as most women will most likely report on their current status, which is less prone to recall bias. Incidence is reported in two periods: from delivery up to and including 3 months post-partum and from 3 to 12 months post-partum and for primi- and multiparous women. STATA Statistical Software, release 15, was used for analysis.

To determine the overall experienced bother in relation to UI across included studies, the measurement results of the different measurement instruments for bother were converted, where possible, to a (standardized) 0 to 100 scale, with 0 indicating no bother and 100 indicating extremely bothered. We classified 1 to 20 as no to mild bother, 20 to 40 as mild to moderate bother, 40 to 60 as moderate to severe, 60 to 80 as severe to very severe and 80 to 100 as extremely severe bother. We used the following conversion method for the ICIQ-UI SF (range 0-21): converted score = observed original score * 4.76 (the value 4.76 is derived from 100 (upper limit converted score)/21 (upper limit original score). Likewise, question 3 from the ICIQ-UI SF (range 0–10) is calculated as follows: converted score = observed original score * 10.

## Results

### Study selection

**Fig. 1:**
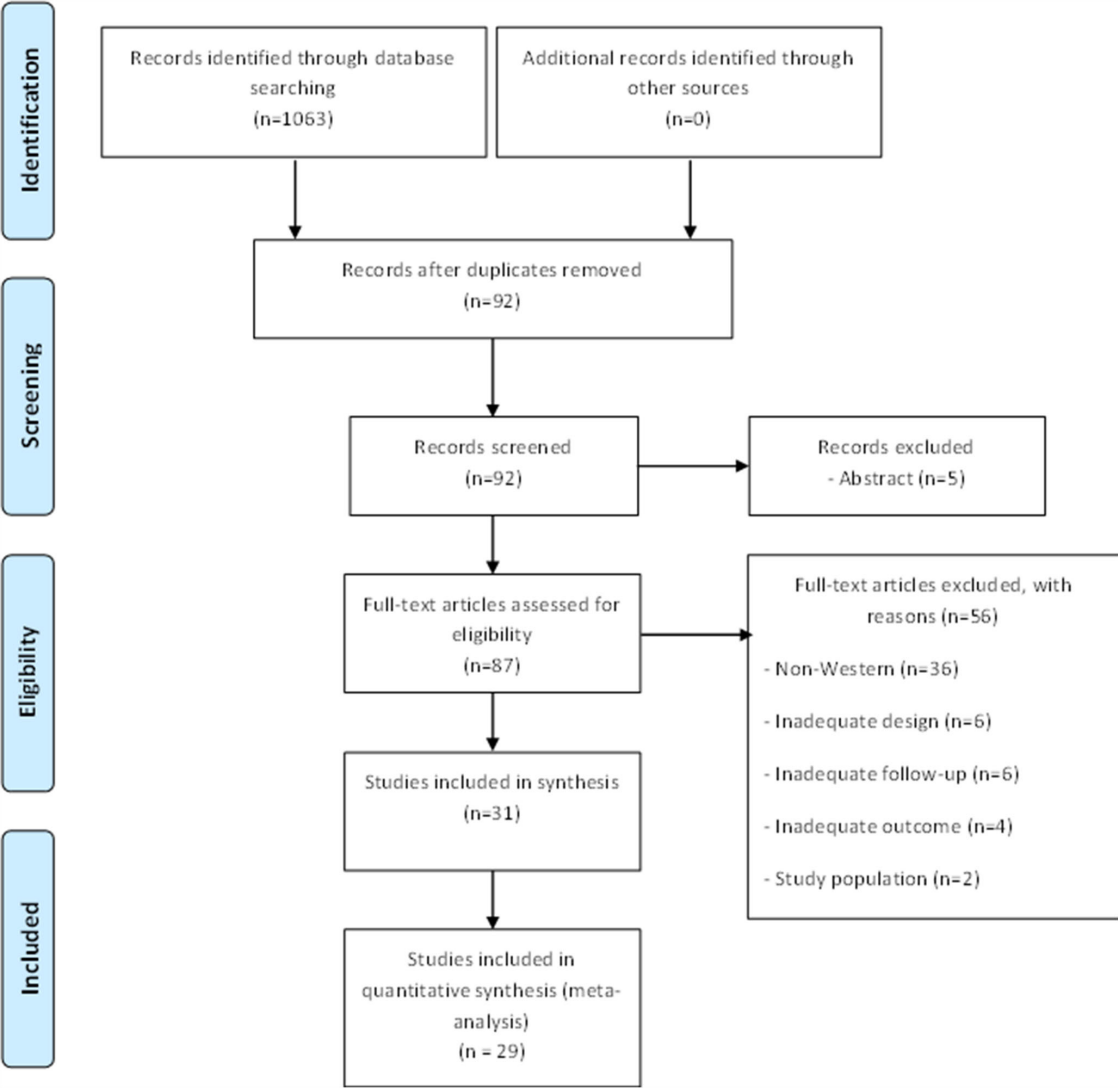
Study flow diagram

Among the 1063 papers initially identified, 31 met the eligibility criteria (Fig. 1), resulting in a total of 38,209 participants. All included studies were observational (20 cohort studies [5, 11, 12, 22–38] and 11 cross-sectional studies [39–49]) and published between 1998 and March 1, 2020. Studies were excluded based on inadequate study design, study population, non-Western countries, outcome, follow-up or language. Twenty-nine studies reported on prevalence and/or incidence figures and two studies only reported on incidence figures.

### Risk of bias

The risk of bias for each study is shown in Table 1. High, moderate and low risk of bias was considered to be present in 1, 26 and 4 studies respectively. Risk-of-bias items with the lowest ratings were 8 and 9, and risk-of-bias items with the highest ratings were 1 and 3.

**Table 1:** Characteristics and outcomes of included studies post-partum

Authors/year	Country	Sample	Case definition UI	Timing measurement(s)	Questionnaire validation	Mean age (years) (SD; range) ^a^	Parity (number of children: n (%)	Sample size (response rate^b^ (%))	Post-partum period	UI prevalence n (%) [incidence: n (%), period]	Type of UI n (%)	Risk of bias items ^c^
Arrue et al. 2010 [39]	Spain	– Primipara – No UI prior to pregnancy Excluded: – Multiple pregnancy – Gestational age < 37 wks – Neurological disorders – Surgery and urogynaecological malformations – Delivery by CS	Urinary leakage on effort (SUI) according ICS terminology	6 months	– Interview – ISI – ICIQ-UI-SF	30.9 (18-43)	Primipara	330 (83.3)	6 months	By type: Incidence (from a term): SUI: 20 out of 227 (8.8)	– SUI: 50 (15.1) – MUI: 10 (3.0) – UUI: unknown –	3,7,8
Baydock et al. 2009 [68]	Canada	– Singleton vaginal delivery Excluded: – UI prior to pregnancy – Drug/alcohol abuse – UI due to medical, cognitive or mobility impairment	Urinary leakage ≥ 1 every 2 weeks	4 months	– Telephone interview – 19-item questionnaire	29 (17-43)	Median: 1 (0-8)	632	4 months	181 (28.6)	By type: SUI: 145 (23.0) UUI: 77 (12.0) MUI: unknown	4,6,7,8,9
Borello-France et al. 2006 [57]	USA	Primiparous women who delivered by caesarean section or vaginally with or without anal sphincter tear Excluded: - IBD - Self-reported prepregnancy anorectal surgery and neurological conditions predisposing to urinary or faecal incontinence	A response of “sometimes” or “often” to any of the MESA questions	6 weeks & 6 months	– Telephone interview – MESA	– Sphinter tear: 27.6 ± 6.0 – Vaginal control: 25.8 ± 5.7 – Caesarean control: 30.2 ± 6.6	Primipara	6 weeks: 837 (91.0) 6 months: 759 (82.0)	6 weeks & 6 months	6 weeks: 282 (33.7) 6 months: 237 (31.2)	By type: 6 weeks: SUI: 128 (45.4) UUI: 37 (13.1) MUI: 117 (41.5) 6 months: SUI: 121 (51.1) UUI: 25 (10.5) MUI: 91 (38.4)	2
Boyles et al. 2009 [56]	USA	Eligible women were identified through Oregon state birth certificates Excluded: Women having abortions, stillbirths or adoptions, out of state residents, and women for whom identifiers were missing	Leakage of urine	3–6 months	Postal self-developed survey	27.7 (5.8)	Primipara	5599	4.4 months (range 3–6)	955 (17.1) [Incidence: 554 (10.0)]	–	2,6,8,9
Brown et al. 1998 [43]	Australia	All women who gave birth in a 2-week period except those who had a stillbirth or known neonatal death	UI not specified	6-7 months	Postal survey	29.4 (15–47)	Primi- & multipara	1366 (62.5)	6–7 months	142 (10.) By parity: - Primiparous: 53 (10.4) - Multiparous: 89 (10.9)	–	6,8
Brown et al. 2015 [69]	Australia	Adult nulliparous women	Leakage of urine at least once per month	3,6,9,12 months	ISI 3,6,12 months: postal survey 9 months: telephone interview	–	Primipara	1507	3,6,9,12 months	-3 months: 416/1415 (29.3) −6 months: 281/1388 (20.2) −9 months: 370/1318 (28.1) −12 months: 356/1348 (26.4) -Any UI: 680/1449 (46.9)		4,5,6,9
Burgio et al. 2003 [5]	USA	Convenience sample, obstetric delivery	Have you ever experienced any difficulty controlling urination? Have you ever had any accidental loss of urine, even a small amount? Have you ever wet yourself?	2–3 days, 6 weeks, 3,6,12 months	2–3 days: face-to-face interview 6 weeks, 3,6,12 months: telephone interview	28.6 (14–42)	Mean: 1.9, median: 2.0 (1–6) Primipara: 41.5%	523 (on 2/3 days)	6 weeks, 3,6,12 months	6 weeks: 56/493 (11.4) 3 months: 45/483 (9.3) 6 months: 47/447 (10.5) 12 months: 51/385 (13.3)		2,6,7,8,9
Chaliha et al. 1999 [23]	UK	Nulliparous women, English-speaking and had singleton pregnancies in the third trimester Excluded: - UTI - History of urinary tract abnormality, recurrent urinary tract infection, history of anorectal surgery or trauma and active anorectal infection	SUI: loss of urine on physical effort or coughing. UUI: loss of urine associated with a strong desire to void	3 months	Structured questionnaire: interviewed in person or by telephone	29 (17–46)	Primipara	549	3 months	80 (14.6)	SUI: 68 (85.0) UUI: 12 (15.0)	6,7,8,9
Cooklin et al. 2015 [12]	Australia	nulliparous, adult pregnant women (singleton, ≥ 36 weeks’ gestation), reporting in pregnancy the intention to breastfeed their infants for at least 8 weeks. Excluded: - Medical conditions prohibitive to breastfeeding; breast reduction surgery; dermatitis on nipple in pregnancy; or requiring services of psychiatrists or social workers during pregnancy.	UI not specified	8 weeks	Telephone interview (week 8 postpartum).	32.6 (4.1; 19–44)	Primipara	222 (73.0)	8 weeks	26 (11.7)		2,3,6,7,8
Dolan et al. 2004 [70]	UK	Primigravidae between 34 and 40 weeks of gestation	Any UI within last 3 months	3 months	Postal self-developed UI questionnaire and KHQ	26 (5.3)	Primipara	362 (73.7)	3 months	47 (13.0)	SUI: 14 (29.8) UUI: 3 (6.4) MUI: 21 (44.7) Missing: 9 (19.1)	6,7,8
Durnea 2014 [11]	Ireland	Nulliparous in their first ongoing pregnancy and having a singleton foetus with a gestational age < 15 weeks. Excluded: preexisting risk factors for pregnancy complications	ICS definitions for urinary dysfunction	1 year	Australian pelvic floor questionnaire	30.5 (4.2)	Primipara	872 (58.8)	1 year	465 (53.9) Incidence: 294 (34.1)	SUI: 204 (43.9) UUI: 72 (15.5) MUI: 189 (40.6) Incidence: SUI: 111 (37.8) UUI: 36 (12.2) MUI: 147 (50.0)	8,9
Farrell 2001 [71]	Canada	– Nullipara – No medical illnesses Excluded: – urinary tract abonormalities or pelvic surgery medication that alters urinary tract function	Do you accidentally lose urine from your bladder?	6 weeks, 6 months	Written self-developed questionnaire	Median 28 (15-48)	Primipara	6 weeks: 559 (94.3) 6 months: 484 (81.6)	6 weeks, 6 months	6 weeks 148/559 (26.5) 6 months: 125/484 (25.8) Incidence: 6 wks: 107/489 (21.9) 6 mo: 89/424 (21.0)	–	6,8
Gartland et al. 2015 [72]	Australia	– ≤ 24 weeks of gestation – Nulliparous, ≥ 18 year – No previous live births or stillbirths at ≥ 20 weeks of gestation	Leaking urine at least once a month	3,6,9,12 months	Postal questionnaire: 3,6,12 months Computer-assisted telephone interviews: 9 months	31.7 (SD 4.6; 19–47)	Primipara	1011 (response rate estimate 28–31%)	0–3, 4–6, 7–9, 10–12 months	0–3 months: 297/1004 (29.6) 4–6 months: 201/992 (20.3) 7–9 months: 282/957 (29.5) 10–12 months: 218/876 (24.9) Period prevalence 0–12 months: 472 (46.7)	0–3 months: SUI: 122 (41.1) UUI: 27 (9.1) MUI: 148 (49.8) 4–6 months: SUI: 114 (56.7) UUI: 26 (12.9) MUI: 61 (30.3) 7–9 months: SUI: 160 (56.7) UUI: 55 (19.5) MUI: 67 (23.8) 10–12 months: SUI: 104 (47.7) UUI: 33 (15.1) MUI: 81 (37.2)	2,5,8,9
Glazener et al. 2006 [55]	Scotland/UK/New Zealand	Primipara Excluded: twin pregnancy	Do you ever lose any urine when you do not mean to?	3 months	Postal self-developed questionnaire	26.7 (5.3)	Primipara	3405 (76.0)	3 months	989 (29.0)	SUI: 459 (46.4) UUI: 221 (22.3) MUI/other: 309 (31.2)	8
Hansen et al. 2012 [27]	Denmark	– Primipara – ≥ 18 years	Any urinary leakage	1 year	– ICIQ-UI-SF – Interference daily life	28.2 (4.8)	Primipara	799 (49.8)	9–12 months	234 (29.3)	SUI: 126 (53.8) UUI: 67 (28.6) MUI: 41 (17.5)	5,8,9
Hatem et al. 2005 [73]	France	Primipara	Score ≥ 2 on the FPSUND tool	6 months	Self-administered questionnaire: FPSUND tool	27.2 (4.8)	1: 1136 (88.0) ≥ 2: 155 (12.0)	1291 (52.0)	171 ± 12 days	382 (29.6)	SUI: 162 (43.0) UUI: 23 (6.0) MUI: 51 (13.0) Unspecified UI: 146 (38.0)	8,9
Huebner et al. 2010 [28]	Germany	– Primipara – Singleton pregnancy – Cephalic presentation – Vaginal delivery – ≥ 38 weeks’ gestation	Do you leak urine involuntarily or does urge lead to UI?	6 weeks & 2 months	Self-developed questionnaire	28.1 (4.7)	Primipara	411 (67.4)	6 weeks & 2 months	6 weeks: 117 (28.5) 2 months: 39 (9.5)	–	6,7,8
Johannessen et al. 2018 [53]	Norway	– Primipara – Healthy infant	Complaint of involuntary loss of urine	12 months	ICIQ-UI SF	UI alone: 29.8 (4.6; 18, 42)	Primipara	976 (65.6)	12 months	382 (39.1)	–	8
Mannion et al. 2015 [74]	Canada	Women who delivered singletons	Since your baby’s birth have you experienced UI (unintentional loss of urine?)	12 months	Self-developed questionnaire	31.5 (4.4)	Primipara: 681 (43.3) Multipara:747 (47.5) Missing: 146 (9.3)	1574	0–12 months	773 (49.1)	–	2,4,8,9
Martin-Martin et al. 2014 [75]	Spain	– Women with singleton pregnancy Excluded: UI before pregnancy	UI not specified	3 and 6 months	Modified ICIQ-UI-SF (by telephone)	31 (5.1)	– 0: 224 (58.8) – ≥ 1: 160 (41.9)	413 (82.6) No previous UI: 381 (76.2)	3 and 6 months	3 months: 43 (11.3) 6 months: 29 (7.6) Incidence 3 months: 13 (30.0)	3 months: SUI: 25 (57.5) UUI/MUI/unclassified: unclear 6 months: SUI predominated	4,7,8
Mason et al. 1999 [32]	UK	Nulli- and multiparous women, regardless of type of delivery	SUI: do you leak urine during physical activity or exertion, for example, whilst coughing, laughing, lifting heavy objects, climbing stairs, during sex etc.?	8–10 weeks	Self-developed questionnaire	Range: 16–45	Primi- and multiparous	572 (64.0)	8 weeks	179 (31.3) Incidence: 45 (7.9)	SUI	4,8
Mørkved et al. 1999 [46]	Norway	All women who delivered at hospital	ICS definition and ‘Do you leak urine at any time: never, seldom, weekly or daily?’	8 weeks	Structured interview 8 wks post-partum	28 (19–40)	$\overline{\times}$: 1.8 (1–5)	144 (72.0)	8 weeks	Self-report: 54 (38.0) Pad test: 28 (19.0) By parity: 1: 21 (38.9) – 2: 19 (35.2) – 3: 11 (20.4) – ≥ 4: 3 (5.6)	–	3,8
Pregazzi et al. 2012 [76]	Italy	Excluded: – Urethra and/or bladder surgery – Lower urinary tract disorders UTI during pregnancy	SUI: UI on physical effort, UUI = UI associated with a strong desire to void	3 months	Face-to-face interviews	19–44	Primipara: 379 (70.7) Multipara: 158 (29.3)	537	3 months	84 (15.6)	SUI: 43 (51.2) UUI: 21 (25.0) MUI: 20 (23.8)	4,8,9
Quiboeuf et al. 2015 [77]	France	– Singleton pregnancy – Excluded: – Women expected to move away from region – Prepregnancy DM	‘Have you had involuntary urinary leakages?’	4 months	-Self-administered postal questionnaire -BFLUTS (type UI) -Sandvik score (severity)	29 (18-44)	Primipara: 493 (30.0) Multipara: 1150 (70.0)	1643 (87.0)	4 months	340 (20.7)	–	4,8
Raza-Khan et al. 2006 [34]	USA	– All pregnant women (3rd trimester) Excluded: – History of preexisting DM – Active cardiac disease excl. mitral valve prolapse – Neurological disease – Urinary tract surgery – Congenital genito-urinary abnormalities	Any MESA answer of ‘sometimes’ or ‘often’	6–8 weeks	- Self-completion written method – MESA – Hunskaar Severity Index	28 (17-41)	– 0: 37 (32.7) – 1: 47 (41.6) – 2: 18 (15.9) – 3: 10 (8.8) – 4: 1 (0.9)	113 (51.6)	Mean: 6.5 weeks Median: 6.3 (SD 1.6)	50 (44.2) Incidence: 5 (4.4)	SUI: 24 (48.0) UUI: 3 (6.0) MUI: 23 (46.0)	3,8,9
Rikard-Bell et al. 2014 [48]	Australia	– Primipara – Non-instrumental delivery Excluded: – Death of baby during labour or post-partum period	UI not specified	≥ 6 months	– Postal questionnaire – PFDI-20-SF – PISQ-12	By group: – Intact perineum: 23.4 (16–41) – Episiotomy: 24.8 (16–38) – Spontaneous tear: 24.4 (15–40)	Primipara	196 (25.6)	≥ 6 months	123 (63.0)	SUI: 62 (50.0)	3,4,5,6,8,9
Schytt et al. 2004 [78]	Sweden	Singleton pregnancy Excluded: women not responding to all 3 questionnaires	UI: Any involuntary loss of urine during the last week SUI: ‘Have you experienced involuntary loss of urine during physical exertion (for example, sneezing or jumping) during the last week?’	12 months	Postal questionnaire	29.5 (median 29.0, SD 4.6)	Primipara: 1051 (44.0) Multipara: 1339 (56.0)	2390 (53.0)	12 months	12 months: SUI: 518 (21.7)	–	2,8,9
Solans-Domènech et al. 2010 [36]	Spain	Healthy nulliparous pregnant women Excluded: – UI before pregnancy – Neurological disease – Cognitive disorders – Urological pathology (non-infectious) – Abortion – Impaired mobility – Previous urogynaecologic surgery – Current treatment with drugs (benzodiazepines, diuretics)	Confirmative answer on ISI (amount/frequency)	7 weeks	– Self-administered during visit – ICIQ-UI-SF – ISI – Effect on dailyliving: VAS (0-10)	–	Primipara	950 (84.2)	7 weeks	155 (16.3) Incidence: 49 (9.0)	SUI: 85 (54.8) UUI: 46 (29.7) MUI: 14 (9.0) Other: 10 (5.8)	4
Thomason et al. 2007 [49]	USA	– Primipara Excluded: – Genital anomalies – Diabetes with risk of UTI – Prior urinary tract infection/surgery – Pregnant – Delivered by CS – UI before pregnancy	Have you involuntarily lost or leaked any amount of urine?	6–9 months	Self-constructed questionnaire	Primiparae continent: 29.7 Primipara incontinent: 29.8	Primipara	121 (75.6)	6 months	52/107 (48.6) Incidence: 2/47 (4.3)	–	3,6,8
Torrisi 2002 [37]	Italy	– Nullipara Excluded: – Previous pelvic surgery – History of recurrent urinary tract infections, women with known malformations of their urinary tract, pre-conceptional hypertension, diabetes, connective tissue disorders, or neurological or cardiological diseases	A score of at least 3 at the ICIQ-SF	3 months	- ICIQ-SF - King’s Health Questionnaire	29.8 (5.6)	Primipara	744 (70.9)	3 months	161 (21.6)	SUI: 61.0 UUI: 15.5 MUI: 12.4 Not reported: 11.1	7,8,9
Wesnes et al. 2007 [79]	Norway	– Primipara – Singleton pregnancy – Continent before and during pregnancy Excluded: – Not completed questionnaire 1, 3 or 4	During coughing/laughing/sneezing, when running/jumping or if they had leakage accompanied by a strong urge to void	6 months	Postal questionnaire	27.9 (4.2)	Primipara	7561	6 months	[Incidence: 1562 (21.0)],	SUI: 651 (41.7) UUI: 471 (30.2) MUI: 440 (28.2)	8,9

^a^Unless otherwise stated

^b^Response rate is based on follow-up assessment in post-partum period (not initial cohort during pregnancy)

^c^Each number represents areas where risk of bias exists (based on the Joanna Briggs critical appraisal tool) [19]. 1 = Was the sample frame appropriate to address the target population? 2 = Were study participants sampled in an appropriate way? 3 = Was the sample size adequate? 4 = Were the study subjects and the setting described in detail? 5 = Were the data analysis conducted with sufficient coverage of the identified sample? 6 = Were valid methods used for the identification of the condition? 7 = Was the condition measured in a standard, reliable way for all participants? 8 = Was there appropriate statistical analysis? 9 = Was the response rate adequate, and if not, was the low response rate managed appropriately?

Incidence defined as new onset UI after delivery

UI = urinary incontinence, IUGA = International Urogynecological Association; ICS = International Continence Society; SUI = stress urinary incontinence, UUI = urgency urinary incontinence, MUI = mixed urinary incontinence, ICIQ-UI-SF = International Consultation on Incontinence Questionnaire-Urinary Incontinence Short Form, IBD = inflammatory bowel disease (ulcerative colitis or Crohn’s disease), wks = weeks, POP = pelvic organ prolapse, IIQ-7 = Incontinence Impact Questionnaire, UDI-6 = Urogenital Distress Inventory, DM = diabetes mellitus, y = years, clin = clinical, Obst = obstetric, UTI = urinary tract infection, inf = infection, QoL = quality of life, I-QoL = incontinence quality of life, PFDI-20 = Pelvic Floor Distress Inventory, ISI = Incontinence Severity Index, QUID = Questionnaire for Urinary Diagnosis, 3-IQ = 3 Incontinence Questionnaire, mths = months, PFDI = Pelvic Floor Distress Inventory, KHQ = Kings Health Questionnaire, sec = second, trim = trimester, MESA = Medical, Epidemiological, and Social Aspects of Ageing Questionnaire, BFLUTS = Bristol Female Lower Urinary Tract Symptom questionnaire; BFLUTS = British Female Low Urinary Track Symptoms, ST = short term, LT = long term

### Study characteristics

The studies originated from Europe (*n* = 17), North America (*n* = 8) and Australia (*n* = 5). One study was mixed (Europe/Australia). The majority of women were included from a (tertiary) hospital (*n* = 26). The remaining studies included women from the community, primary health care service or health care insurance service. Nineteen studies only reported on primiparous women. Twelve studies used validated questionnaires to determine the presence of UI, and 19 studies used self-constructed, non-validated questionnaires. Table 1 summarizes the study characteristics of included studies.

Nine studies reported on (measurement instruments for) bother. Table 2 provides an overview of the measurement instruments as used in the included studies, with the original and the converted (0–100 scale) measurement results. Five different measurement instruments for bother were used, of which the ICIQ-UI SF was most frequently used [27, 29, 37, 39]. One study only reported the results of the ICIQ-UI SF as categories [50], and two studies did not report total scores [24, 45]. One measurement instrument was self-constructed and non-validated [30].

**Table 2:** Measurement of bother and results

Measurement instrument	Background information on measurement instrument	Study	Original measurement result (mean)	Period post-partum	(Converted) measurement results (0–100)
ICIQ-UI SF (0-21)	To assess symptoms of UI and impact on QoL. (4 questions, question 4 is on moment of UI and is not within the calculation of the total)	[39]	8.2 for SUI	6 months	39.0
			10.0 for MUI	6 months	47.6
		[27]	5.9	1 year	28.1
		[53]	5.1	1 year	24.3
		[37]	6.0	3 months	28.6
		[50]	Results reported in categories. No total score		
ICIQ-UI SF Question 3 (QoL) (0-10)	Question 3 of the ICIQ-UI SF is on the interference in daily life of UI	[31]	4.1	3 months	41.0
			4.5	6 months	45.0
I-QOL	Quality of life in persons with UI. 3 subscales: 1. Avoidance and limiting behaviour, 2. Psychosocial impact, 3. Social embarrassment (22 questions)	[45]	No exact scores reported		
KHQ		[24]	No total score reported		
Self-constructed questionnaire		[30]	No total score reported		

ICIQ-UI-SF = International Consultation on Incontinence Questionnaire-Urinary Incontinence Short Form, QoL = quality of life, I-QoL = incontinence quality of life, KHQ = Kings Health Questionnaire, SUI = stress urinary incontinence, MUI = mixed urinary incontinence

### Synthesis of results

#### Overall prevalence

**Fig. 2:**
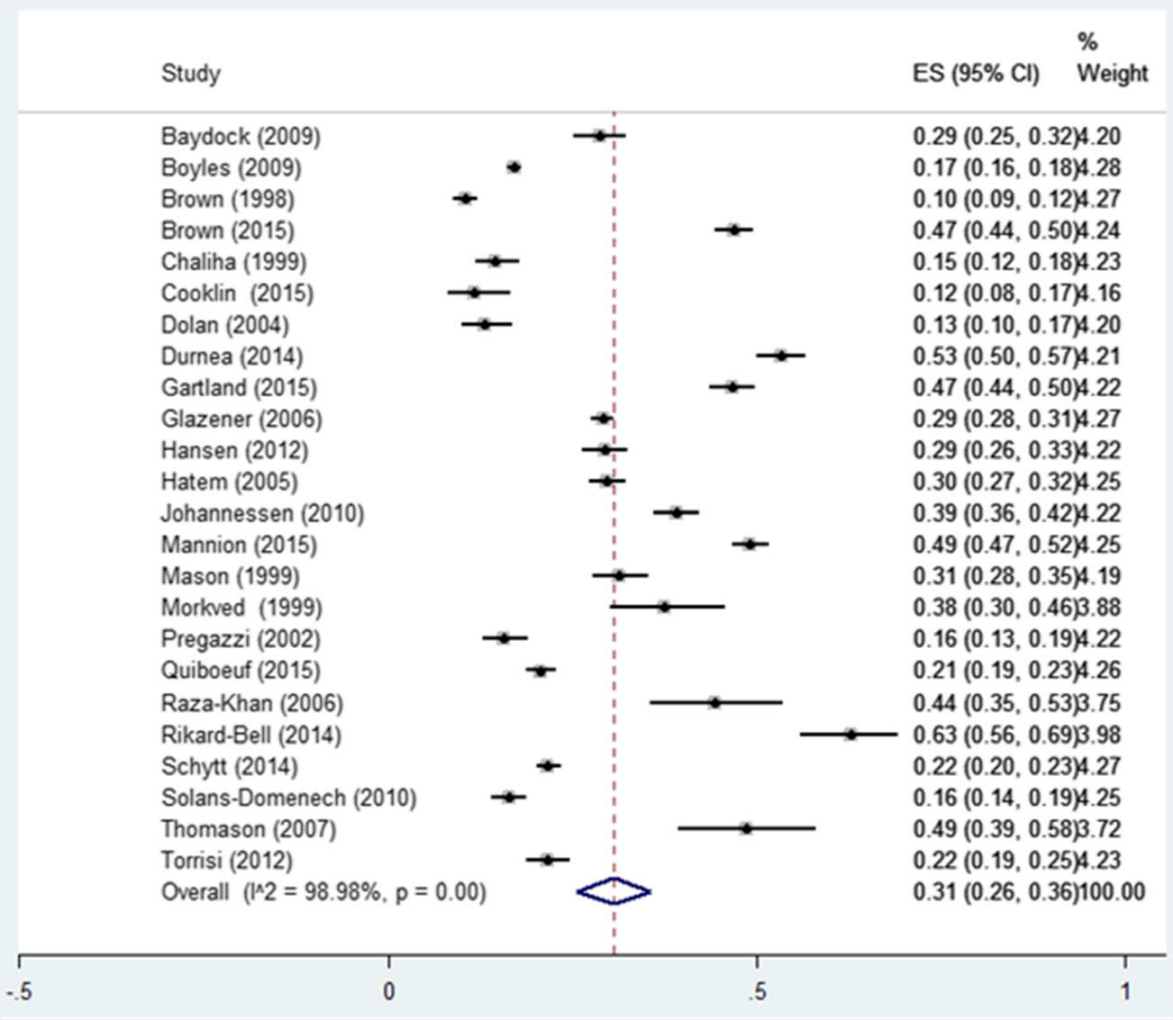
Pooled prevalence of UI post-partum

Twenty-four out of 31 studies contributed to the calculation of the overall prevalence of post-partum UI, involving a total of 35,064 women. The weighted mean of UI prevalence among post-partum women (6 weeks to 12 months) was 31.0% (CI 95% 26.0–36.0%; I^2^: 99.0%), regardless of parity or type of UI (Fig. 2). The lowest prevalence of UI found in the included studies was 10% [51] and the highest prevalence 63% [52]. Prevalence figures for studies with low (*n* = 3), moderate (*n* = 20 studies) and high risk of bias (*n* = 1) were 28% (95% CI 17.0–39.0), 29% (95% CI 24.0–35.0) and 63%, respectively (Table 1).

#### Subcategories post-partum period, type of UI and parity

**Fig. 3:**
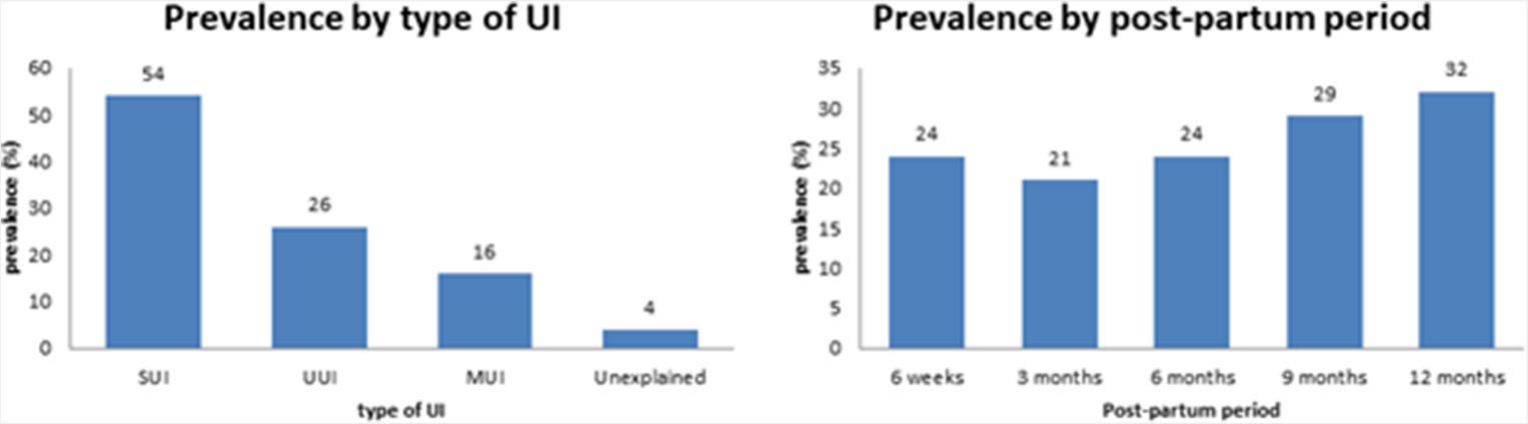
Prevalence of UI by type and period

Figure 3 summarizes the mean UI prevalence at 6 weeks, 3, 6, 9, and 12 months post-partum. From an initial drop in (weighted) prevalence between 6 weeks [24.0%, 95% CI: 17.0-32.0% (1349/5137)] and 3 months post-partum [21.0%, 95% CI: 17.0–25.0% (3677/17,165)], prevalence numbers gradually rise to 32.0% at 12 months post-partum (95% CI: 23.0–41.0% (2997/9220)). The prevalence of UI post-partum is equal among primi- and multiparous women, 31% [11, 12, 22–24, 26, 27, 36, 37, 42, 44, 48, 49, 53] and 30% [30, 33–35, 40, 43, 45–47, 54], respectively.

Based on nine studies, SUI accounts for 54% UUI and MUI for 26% and 16% of cases respectively, whereas 4% was unexplained UI [23, 24, 27, 34, 36, 37, 44, 47, 48].

#### Subcategories frequency and amount of UI

Seven out of 31 studies reported on frequency of UI. The most used frequency categories (*n* = 3) were: less than once a week, less than daily, more than or equal once per week and more than or equal daily leakage. A frequency of less than once a week was most frequently reported (50%–66.3%) [23, 46, 55]. Two studies reported frequency of UI as: less than once per month, a few times a month, a few times a week, every day and/or night [34, 50]. One study reported: occasionally, once per week, several times per week and daily [32] and one study reported the ICIQ-UI SF question on frequency [27].

Four studies reported on the amount of urine loss [5, 27, 34, 50]. One study used the ICIQ-UI SF to assess this parameter (none, small, moderate, large amount) [27]. One study reported the ICIQ-UI SF item ‘amount’ separately, showing that the majority of UI patients lose a small amount (85.3%) [27]. Other descriptions of amount of urine lost used were: drops, small splashes and more [34, 50]. Drops were most frequently reported in 71.6% of cases [50]. The remaining study reported amount as a drop or two, pad or clothing damp, and pad or clothing soaked [5].

#### Bother

Nine studies reported on impact on daily life or quality of life [24, 27, 30, 31, 37, 39, 45, 50, 53], which was heterogeneously assessed. The ICIQ-UI SF total score was used most frequently (*n* = 5). Martin-Martin et al. reported the impact on daily life (0–10) based on the ICIQ-UI SF [31]. Other questionnaires used once to assess impact on daily life were: Incontinence Quality of life (I-QOL) [45], King’s Health Questionnaire (KHQ) [24] and a self-constructed non-validated questionnaire [30]. The overall bother of UI post-partum, on a 0 to 100 scale, ranges between 24.3 and 47.6, consistent with mild to moderate bother. At 3 months post-partum, degree of bother ranged between 28.6 and 41.0, at 6 months post-partum between 39.0 and 45.0 and at 12 months post-partum between 24.3 and 28.1 (Table 2).

#### Case definition

The majority of studies (*n* = 11) used ‘any leakage’ as a case definition [5, 24, 25, 27, 28, 30, 33, 49, 53, 55, 56]. Eight studies used the ICS definition, which was not mentioned as such in some cases [11, 23, 32, 35, 38, 39, 46, 47]. Six studies did not specify a case definition for UI [12, 31, 37, 43, 48, 57], five used a frequency [22, 26, 34, 40, 50], and one study used the Clinical Classification of Urinary Incontinence (FPSUND) [45].

#### Incidence

Ten studies have examined the incidence of UI post-partum (Table 1) [11, 25, 31, 34, 36, 38, 39, 49, 54, 56]. Five studies reported incidence up to and including 3 months [25, 31, 34, 50, 54] and six reported from 3 until 12 months [11, 25, 38, 39, 49, 56]. One study reported for both periods [25]. The incidence of UI in primiparous and multiparous women up to 3 months was 9.0–21.9% and 4.4–30.0%, respectively. Incidence up to 1 year was 4.3–34.1% in primiparous women.

## Discussion

The aim of this systematic review and meta-analysis was to summarize the pooled prevalence and incidence of UI between 6 weeks and 12 months post-partum, to provide an overview of assessment methods for bother in relation to UI and to assess the degree of bother post-partum. The results show an overall mean prevalence rate of UI up to 1 year post-partum of 31%, with a range of 10% to 63%. The prevalence of 10% was reported in a study on maternal health using a generic questionnaire including only one question on UI [43] in contrast to the other studies using health problem-specific questionnaires. This might have influenced the tendency for women to report UI [58].

The prevalence numbers in the first year post-partum rise from 24% at 6 weeks to 32% at 12 months post-partum after an initial drop between 6 weeks and 3 months. A recently published systematic review and meta-analysis on the prevalence of UI during pregnancy reported a prevalence of UI of 34% in the third trimester [59]. The drop in UI prevalence early post-partum compared to the third trimester of pregnancy might be explained by the natural recovery of the levator ani muscle, which occurs mainly up to 4 to 6 months post-partum [60, 61]. The rise in prevalence from 3 to 12 months post-partum might be due to return to daily activities, such as return to work and starting with sports, with an associated increase in physical activity level and as a consequent loading of the continence system [62, 63]. The prevalence of UI between primi- and multiparous women was nearly equal (31% and 30%). This is in line with the EPINCONT study on 27,900 women, which reported that the first delivery is the largest risk factor for UI, more specifically SUI and MUI, post-partum [64].

Thom et al. published a systematic review with 33 studies on the prevalence of post-partum urinary incontinence. The overall prevalence reported by Thom et al. between 2 and 13 weeks post-partum was 33.3%. As only one included study covered the period of 14 to 52 weeks post-partum, an overall prevalence number could not be calculated [13]; 33.3% is a higher prevalence than the 24% at 6 weeks and 21% at 3 months reported in this study. This might be due to the fact that Thom et al. did not report a weighted prevalence.

When interpreting the prevalence numbers at different time points post-partum, it is important to keep in mind that UI might be a dynamic phenomenon. This means that a woman’s continence status can change both ways over a period of time [33].

The incidence numbers between 6 weeks up to 3 months and 3 months up to 12 months and among primi- and multiparous women varied. The low incidence number of 4.3% in the short term might be explained by the fact that this study only reported on SUI or MUI incidence [49]. Although the study of Thomason et al. claims to report the incidence of total UI, only women who reported UI with a positive (cough) stress test were included. Women who were able to contract their pelvic floor muscles properly and timely during an anticipated known rise in abdominal pressure might therefore be considered continent. However, these women might be incontinent during an unexpected rise in abdominal pressure. Also, the small sample (*n* = 121) this study is based on might distort the results. If the overall incidence of the up to 3 months post-partum is compared with the up to 12 months post-partum group, the incidence numbers show a small rise in the latter, 4.3–30.0% and 4.4–34.1%, respectively. The rise in incidence follows the pattern of the rise in prevalence of UI between 3 and 12 months post-partum.

Most included studies showed a moderate risk of bias, which influences the possibility to differentiate prevalence between groups regarding risk of methodological quality. The mean prevalence of UI reported by studies with low and moderate risk of bias did not differ. However, the one high risk study reported the highest prevalence of 63% [48]. Because a weighted prevalence number was calculated, this high risk study with only 196 participants and low response rate of 25.6% hardly influences the overall prevalence of UI.

The ICI recommends reporting prevalence numbers along with a measure of experienced bother [14]. Only 9 out of 31 studies (approximately 30%) reported bother in relation to UI with a variety of measurement instruments, which shows that combined assessment is not yet common practice [24, 27, 30, 31, 36, 37, 39, 45, 53]. Eight studies used high-quality measurement instruments, most frequently the ICIQ-UI SF [27, 36, 37, 39, 53]. In an attempt to provide an overall assessment of degree of experienced bother in relation to UI, after studying all available materials, we decided to standardize the measurement results of different bother scales to a 0 to 100 scale. The 0 to 100 scale can be regarded as a visual analogue scale (VAS). The VAS is a valid and reproducible method to quantify the impact of UI on QoL [65], although no studies are known that report on cut-off scores for QoL specifically in post-partum women with UI. Boonstra et al. compared the VAS with a measure that assesses the impact on functioning in patients with pain and identified three classes: class 1, mild interference (score 1–34), class 2, moderate interference (score 35–64) and class 3, severe interference with daily life (score 65–100) [66]. Based on these classes, this systematic review revealed that women experience their post-partum UI as mild to moderate (range 24.3–47.6). Based on two studies, the results show a trend that bother of UI reduces at 12 months post-partum [27, 53]. Women report for instance that UI becomes less of a problem because they get used to it and that they find practical ways to cope by using panty liners and avoiding certain activities [54].

Nevertheless, over half of the women with UI post-partum think that it will improve by itself in time and only 25% of women with post-partum UI actually seek help [67]. However, 73% of women with UI 3 months post-partum still report UI at 6 years post-partum [6]. Reliable information on UI prevalence is thus essential to estimate health care burden, allocation of health care resources and research planning.

### Strengths and limitations

The strength of this systematic review and meta-analysis is the large number of included studies, which resulted in the availability of prevalence and incidence numbers for different subpopulations (parity, post-partum period, type of UI) and for different purposes (health care providers, research planning and policy makers). This is the first review to report the prevalence and incidence over the first 12 months post-partum and bother in relation to post-partum UI.

The limitations of this study are, first, the presence of substantial clinical heterogeneity of the studies. Clinical heterogeneity may be due to differences in: case definition (any UI or different frequencies of UI in a certain period of time), population (primiparous and multiparous) or periods researched. Second, the considerable statistical heterogeneity of the studies resulted in large CIs. Third, as the Joanna Briggs critical appraisal tool does not recommend cut-off points for high, moderate or low risk of bias, we arbitrarily chose the cut-off points reported in this systematic review to explore possible differences in prevalence numbers if stratified for risk of bias. However, we did not include or exclude studies based on risk of bias.

## Conclusion

After an initial drop in prevalence of UI at 3 months post-partum (21%), at 1 year post-partum, prevalence rises again to 31%. UI prevalence does not differ between primi- and multiparous women. Bother of UI is heterogeneously assessed and is reported as mild to moderate.

## Supplementary Information

ESM 1 (DOCX 12 kb)
