# Supplementary material for: Prevalence, incidence and bothersomeness of urinary incontinence between 6 weeks and 1 year post-partum: a systematic review and meta-analysis
Source: Int Urogynecol J. 2021 Jun 17;32(7):1675–93. doi: 10.1007/s00192-021-04877-w (PMC8295150; doi:10.1007/s00192-021-04877-w)
Supplement: Supplementary file 1 — (DOCX 12 kb) [file 192_2021_4877_MOESM1_ESM.docx]

**Supplement 1:**

**Search strategy for PubMed:**

**Appendix 1:**

(((((((((((('urinary incontinence'[MeSH Terms]) OR urinary incontinence title/abstract) OR 'urine loss'[Title/Abstract]) OR 'pelvic floor disorders'[MeSH Terms]) OR 'pelvic floor disorders'[Title/Abstract]) OR 'pelvic floor dysfunctions'[Title/Abstract])) OR incontinence[Title/Abstract])) OR 'leaking urine'[Title/Abstract])) AND ((((((((pregnancy[MeSH Terms]) OR pregnancy[Title/Abstract]) OR pregn[Title/Abstract])) OR (((((((((postpartum[Title/Abstract]) OR post-partum[Title/Abstract]) OR post partum[Title/Abstract]) OR postpartum[Title/Abstract]) OR post-partum[Title/Abstract])) OR peripartum[Title/Abstract]) OR peri-partum[Title/Abstract]) OR peri partum[Title/Abstract])) AND ((((nulliparous[Title/Abstract]) OR primiparous[Title/Abstract]) OR primigrav*[Title/Abstract]) OR primipara[Title/Abstract]))))) AND ((((((((((((((((((((((((prevalence[MeSH Terms]) OR prevalence[Title/Abstract]))) OR epidemiology[MeSH Terms])) OR epidemiology[Title/Abstract]) OR quality of life[MeSH Terms]) OR 'quality of life'[Title/Abstract]) OR bother*[Title/Abstract]) OR bothersomeness[Title/Abstract]))
